# A novel binding site on the cryptic intervening domain is a motif-dependent regulator of O-GlcNAc transferase

**DOI:** 10.21203/rs.3.rs-2531412/v1

**Published:** 2023-02-02

**Authors:** Connor Blankenship, Jinshan Xie, Caroline Benz, Ao Wang, Ylva Ivarsson, Jiaoyang Jiang

**Affiliations:** University of Wisconsin-Madison; University of Wisconsin-Madison; Uppsala University; University of Wisconsin-Madison; Uppsala University; Pharmaceutical Sciences Division, School of Pharmacy, University of Wisconsin-Madison

## Abstract

The modification of intracellular proteins with O-linked β-*N*-acetylglucosamme (O-GlcNAc) moieties is a highly dynamic process that spatiotemporally regulates nearly every important cellular program. Despite its significance, little is known about the substrate recognition and regulation modes of O-GlcNAc transferase (OGT), the primary enzyme responsible for O-GlcNAc addition. In this study, we have identified the intervening domain (Int-D), a poorly understood protein fold found only in metazoan OGTs, as a specific regulator of OGT protein-protein interactions and substrate modification. Utilizing an innovative proteomic peptide phage display (ProP-PD) coupled with structural, biochemical, and cellular characterizations, we discovered a novel peptide motif, employed by the Int-D to facilitate specific O-GlcNAcylation. We further show that disruption of Int-D binding dysregulates important cellular programs including nutrient stress response and glucose metabolism. These findings illustrate a novel mode of OGT substrate recognition and offer the first insights into the biological roles of this unique domain.

## Introduction

Regulation of intracellular primary metabolites is a delicate balance between excess and deficit, each of which can have deleterious effects on the health of a cell. One major signaling corridor for cellular nutrient sensing is the hexosamine biosynthetic pathway (HBP), which uses amino acids, nucleotides, carbohydrates, and fatty acids to produce uridine diphosphate *N*-acetylglucosamine (UDP-GlcNAc)^1^. This allows the level of each essential metabolite to influence HBP flux and cellular concentration of the nucleotide sugar. In turn, UDP-GlcNAc serves as the activated sugar donor of O-GlcNAc transferase (OGT), the single enzyme responsible for most mono-O-linked β-*N*-acetylglucosamine addition (O-GlcNAcylation) on serine and threonine residues of intracellular proteins across all major cellular processes^2^. Since O-GlcNAcylation dynamically and sensitively regulates numerous highly coordinated processes including the cell cycle^3^, gene expression^4^, and proteasomal degradation^5^, this modification has been proposed to be a central nutrient sensing system^6,7^. Unlike many other enzymes, OGT does not recognize an apparent sequence motif at the modification site, and little is known about this enzyme’s substrate recognition and regulation modes.

Coordination of the highly spatiotemporally dynamic cellular environment requires enzymes to be in the right place at the right time to interact with their targets. For many enzymes, particularly those that recognize a wide breadth of protein substrates, this is achieved by association with larger protein machinery or binding to adaptor proteins through specific protein-protein interactions (PPIs)^8^. Often these interactions are facilitated through scaffolding domains or discrete binding sites, outside of the enzyme catalytic pocket, that recognize short linear motifs (SLiMs)^9–11^. However, these binding interactions can be difficult to map as they are typically low affinity and facilitated by poorly defined shallow grooves. It is therefore necessary to apply new technology to both identify and understand the complex regulatory networks that direct essential cellular proteins.

**Figure 1:**
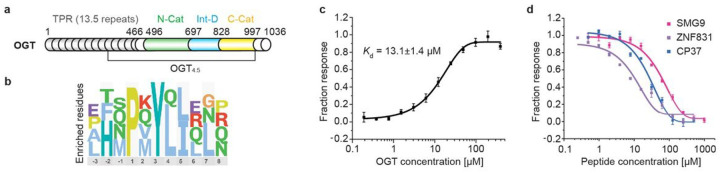
ProP-PD screening identified a specific OGT binding motif. (**a**) Domain schematic of full-length OGT with tetratricopeptide repeat (TPR) domain in gray, N-terminal catalytic (N-Cat) domain in green, intervening domain (Int-D) in blue, and C-terminal catalytic (C-Cat) domain in yellow. The crystallization construct OGT_4.5_ with only 4.5 of 13.5 TPR repeats was also shown. (**b**) Sequence logo of highly enriched peptides from both OGT and OGT_4.5_ ProP-PD screens, aligned to the PxYx[I/L] motif. (**c**) Microscale thermophoresis (MST) binding assay of SMG9 peptide with OGT, n=3. (**d**) Competitive fluorescence polarization (FP) binding assay with fluorescently labeled 5-FAM-SMG9 peptide competing with unmodified SMG9, ZNF831, and consensus peptide 37 (CP37), for OGT binding, n=3. Error bars represent standard deviation of three replicates.

OGT is a multidomain enzyme consisting of 13.5 tetratricopeptide repeats (TPRs) and a catalytic region (Fig. 1a)^12–14^. Tandem TPR motifs are commonly found in protein binding scaffolding, directing the focus of many studies toward identifying the TPR domain’s role in substrate recognition and protein binding. Our group and others have identified key TPR residues, including parallel aspartate and asparagine ladders, that facilitate OGT substrate binding^15–19^. Specific protein substrates have also been studied to map interacting residues on the TPR domain showing that even TPRs distal to the OGT catalytic site may be involved in substrate recognition^18,19^. Despite these efforts, a full model of OGT substrate recognition and its connection to the O-GlcNAc regulatory network remains obscure.

The OGT catalytic region is comprised of twin N- and C-catalytic lobes (N-Cat and C-Cat) separated by an intervening domain (Int-D) (Fig. 1 a). Both catalytic lobes have Rossmann-like structures, typical of the GT-B superfamily of glycosyltransferases^12^. However, human OGT and its homologs in metazoans are the only known proteins to contain an Int-D. Since its identification over a decade ago, the function of the Int-D has remained mysterious. To advance our understanding of this essential enzyme and its roles in the cell, we employed an innovative proteomic peptide phage display (ProP-PD) to profile PPI sites across the surface of OGT^20^. Using X-ray crystallization and biochemical approaches, we identified a novel binding site in the OGT Int-D that was revealed to be a motif dependent regulator of OGT binding and substrate specific O-GlcNAcylation. This is the first evidence of SLiM-based OGT substrate recognition and the first discrete binding site identified in the unique Int-D. Moreover, we uncovered a mode of indirect posttranslational modification (PTM) crosstalk through tyrosine phosphorylation of our novel motif. Revealing the specific binding site and mode of PTM crosstalk gives the first structurally justifiable mechanism of cellular communication between phosphorylation and O-GlcNAcylation. We further demonstrate the surprising roles of this binding site in regulating the O-GlcNAcylation response to nutrient stress as well as lactate production. In addition to current hypotheses that propose HBP flux as a key driver of O-GlcNAcylation nutrient sensing^21^, we demonstrate that mutation of the Int-D can significantly delay the O-GlcNAcylation response to nutrient starvation. These findings reveal important roles of the OGT Int-D that has garnered interest for its unique character but to this point remained cryptic.

## Results

### ProP-PD identified a novel OGT binding motif

To directly identify biologically relevant OGT peptide binders, we employed an innovative phage display flatform. The unique Proteomic Peptide Phage Display (ProP-PD) library of overlapping 16-mer peptides is designed around intrinsically disordered regions of the human proteome^20,22^. Compared to commonly used phage display platforms, ProP-PD features higher peptide copy number (~ 200 per virion) through display on the major coat protein P8 of the filamentous M13 phage, which enables enrichment of moderate to low affinity binders through avidity^23^. The targeted design also allows us to only screen biologically relevant peptides that map to human proteins. This leads to identification of SLiMs that facilitate protein-protein interactions at amino acid resolution, which is difficult to obtain using traditional experimental approaches. We screened the ProP-PD library against two OGT constructs, full-length OGT (OGT) and the protein crystallization construct OGT_4.5_ (Fig. 1a). When recombinantly expressed and purified, these protein constructs are well folded and highly stable, allowing us to evaluate whether peptides bind to the TPR or catalytic region based on their enrichment by one or both constructs. Enriched phage pools were sequenced with next-generation sequencing (NGS) and peptides were considered as hits if they appeared in multiple replicates or if two overlapping sequences were identified. Remarkably, both OGT constructs yielded a similar peptide population with a novel strongly enriched motif PxYx[I/L] (Fig. 1 b) identified by the SLiMFinder algorithm^24,25^, which to our knowledge has not previously been described^11^. Of the 24 most highly enriched peptides in the NGS dataset, 16 contain this exact motif or a single amino acid variant. Enrichment of a single motif, by OGT and OGT_4.5_, suggests this is the dominant peptide binding site localized to the OGT catalytic region. However, the absence of Ser/Thr in the motif implies that this is not a direct active site binder. Seven natural peptides and one consensus peptide (CP37) were selected for synthesis and further characterization (Extended data Fig. 1 a).

We began assessing peptide interactions with OGT using the thermal shift assay (TSA), a rapid label-free binding assay that has been widely used to evaluate target-ligand interactions by measuring changes in protein denaturation temperature (T_m_)^26^. All but one peptide showed a significant T_m_ increase with both OGT and OGT_4.5_ (Extended data Fig. 1b). We note that some of our identified peptides mapped to proteins with well-characterized biological functions or known links to O-GlcNAcylation. For instance, SMG9 protein is a critical component of cellular nonsense mediated mRNA decay (NMD), a process that targets transcripts with premature stop codons for degradation^27^. Notably, SMG9 has a single major O-GlcNAcylation site at residue T114, located 30 amino acids upstream of the identified motif, making SMG9 an interesting target to investigate further^28,29^. Applying a fluorescently labeled 5-FAM-SMG9 peptide, we measured its binding affinity to OGT by microscale thermophoresis (MST)^30^. This binding assay showed a clear dose-response relationship and produced a *K*_d_ of 13.1 mM (Fig. 1 c), which would be considered a moderately strong SLiM PPI. As our synthesized peptides contain a single conserved motif, we hypothesized that they were all binding to a single site on OGT. The remaining unlabeled peptides were evaluated by competitive fluorescence polarization (FP) assay with fluorescently labeled 5-FAM-SMG9 peptide (Extended data Fig. 1). Top binders (ZNF831 and CP37) demonstrated strong dose-response relationships with apparent binding affinity (EC_50_) similar to the SMG9 peptide (Fig. 1 d, Extended data Fig. 1 a).

### Co-crystallization Revealed A Novel Binding Site In The Ogt Int-d

**Figure 2:**
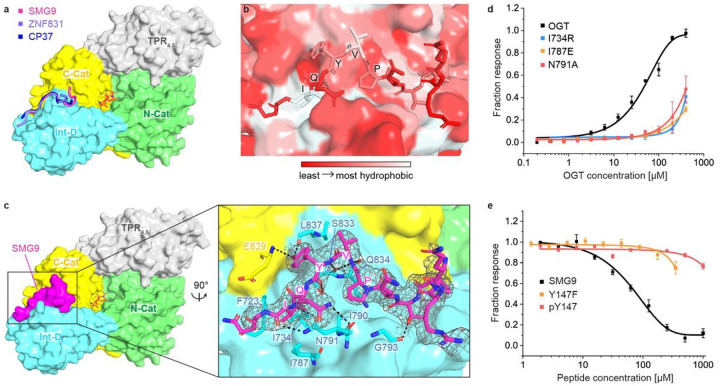
OGT crystal structures reveal a novel binding site in the Int-D. (**a**) OGT_4.5_ in complex with Int-D-binding peptides and UDP-GlcNAc. SMG9, ZNF831, and CP37 peptides are shown as cartoon in magenta, purple and blue, respectively. Protein domains in OGT_4.5_ are colored as in Figure 1 a. UDP-GlcNAc is shown in orange sticks. (**b**) Zoom-in view at the Int-D binding site demonstrating hydrophobic interactions between SMG9 peptide (shown as sticks) and Int-D in OGT_4.5_ (shown as surface). Red to white color scale represent hydrophobicity. (**c**) Left: surface representation of SMG9 peptide bound to OGT_4.5_. Right: zoom-in view at the Int-D binding site demonstrating polar interactions between SMG9 peptide (shown as magenta sticks) and Int-D in OGT_4.5_ (shown as surface) with interacting OGT residues shown in sticks. 2*F*_o_-*F*_c_ electron density map of SMG9 peptide is shown as grey mesh and contoured at 1.0 σ. (**d**) Saturation fluorescence polarization (FP) binding assay of fluorescently labeled 5-FAM-SMG9 peptide with wild-type (WT) OGT (black) and mutants I734R (blue), I787E (orange), and N791A (red), n=3. (**e**) Competitive FP binding assay of WT SMG9 (black), mutant SMG9 Y147F (orange) and phosphorylated SMG9 pY147 (red) peptides with fluorescently labeled 5-FAM-SMG9 peptide binding to OGT, n=3. Error bars represent standard deviation of three biological replicates.

Capitalizing on our expertise in protein crystallization, we sought to obtain structural insights into these novel interactions. We co-crystallized OGT_4.5_ with each of the three top binding peptides (SMG9: 140–155 aa, ZNF831: 928–943 aa, and CP37) in the presence of UDP-GlcNAc. We successfully determined the OGT_4.5_:UDP-GlcNAc:SMG9 and OGT_4.5_:UDP-GlcNAc:CP37 structures at 2.98 and 3.06 A, respectively, while the OGT_45._:UDP-GlcNAc:ZNF831 structure was determined at 3.69 Å (Extended data table 1 and Extended data Fig. 2a-d). Strikingly, unambiguous electron densities clearly show that all three peptides bind to the OGT Int-D in a highly conserved conformation (Fig. 2a, c and Extended data Fig. 2b, d). We note that the peptide binding did not induce significant change to the OGT_4.5_:UDP-GlcNAc structure (Extended data Fig. 2e). Given the high similarity of these peptide binding conformations, we focus on the OGT_4.5_:UDP-GlcNAc:SMG9 structure in our following discussions.

In the complex structure, SMG9 peptide binds in an elongated conformation in a shallow Int-D surface groove, displaying favorable shape complementarity (Fig. 2a, c). Peptide binding leads to 1,247 Å^2^ buried surface area, which is in the typical range for transient PPIs^31,32^. We observed strong electron density for the SMG9 peptide backbone and most of its side chains, allowing us to analyze this interaction with high confidence. The binding conformation of the SMG9 peptide is mainly supported by two types of interactions: hydrophobic (Fig. 2b) and hydrogen bonding (Fig. 2c). OGT Int-D site binding specificity is likely driven by hydrophobic interactions. The motif isoleucine side chain of SMG9 peptide (corresponding to the I149 residue of SMG9 protein) reaches into a small hydrophobic pocket formed by highly conserved OGT residues I734, I787 and F723. Additionally, the SMG9 motif proline (P145) residue interacts with hydrophobic I790, lining the Int-D surface groove, allowing appropriate positioning of the peptide motif. Besides these hydrophobic features, OGT Int-D N791 also plays critical roles in stabilizing peptide binding through polar interactions. The carboxamide side chain of N791 makes bidentate interactions with the backbone of SMG9 I149. The N791 backbone carbonyl oxygen further accepts a hydrogen bond from the carboxamide sidechain of Q148 in the SMG9 peptide. Another indispensable element in this interaction is the SMG9 motif tyrosine (Y147) residue. The side-chain hydroxyl of SMG9 Y147 forms double hydrogen bonds with OGT L837 and E839 backbones, while the Y147 backbone makes two additional hydrogen bonds to OGT S833. Moreover, the interactions between backbones of SMG9 V146 and OGT S833/Q834 further stabilize the peptide binding conformation. Outside of the motif, SMG9 T143 side chain mediates a hydrogen bond with the backbone carbonyl of OGT G793. Collectively, these hydrophobic and hydrogen bonding interactions form a tangled network stabilizing the binding of SMG9 peptide in the OGT Int-D.

To confirm the SMG9 peptide binding mode in our crystal structure, we mutated each of the two primary binding features: the hydrophobic pocket (F723, I734, and I787) and the bidentate donor N791, and measured their changes in binding affinity for SMG9 peptide. Each of the hydrophobic OGT residues were mutated to a glutamate or arginine, while the asparagine bidentate interaction was disrupted by mutation to alanine. Each mutated OGT construct was recombinantly expressed in E *coli* and purified at high concentration for FP saturation binding assays with fluorescent SMG9 peptide. Insertion of charged residues into the hydrophobic pocket (F723E, I734R, I787E, and I787R) as well as disruption of the bidentate backbone interaction (N791 A) each shifted EC_50_ values by >10-fold (Fig. 2d, Extended data Fig. 3a), showing that these mutations substantially disrupt SMG9 peptide binding. TSA of each OGT mutant showed no or slight changes in protein stability (Extended data Fig. 3b). These results strongly support the peptide binding mode illustrated in our crystal structure and offer useful mutants for interrogation of this binding site’s role in cells.

### Smg9 Y147 Phosphorylation Disrupts Peptide Binding To Ogt

On the other side of this interaction, the SMG9 Y147 residue is highly conserved in our motif and makes substantial interactions with OGT Int-D (Fig. 2c), signaling its importance for binding. To further confirm the SMG9 binding mode observed in our crystal structure, we introduced a Y147F mutation to the SMG9 peptide. Using the same FP competition assay as before, SMG9 Y147F demonstrated > 10-fold shift in EC_50_ (Fig. 2e), supporting the significant role of motif Y side chain in OGT interactions. Interestingly, Y147 is one of the major phosphorylation sites on SMG9 protein. Previous studies have linked phosphorylation at this site to transcriptional programs in epidermal growth factor signaling^29^. Structural analysis of this modification on Y147 suggests that it would not be tolerated by the Int-D binding site. Using our FP competition assay, we tested the interaction of phosphorylated SMG9 peptide (pY147) with OGT. SMG9 pY147 significantly disrupted peptide binding, to a similar degree as Y147F (Fig. 2e). Tyrosine phosphorylation (pTyr) is a fundamental mechanism of cellular regulation^33^. Compared to the much more prevalent Ser/Thr phosphorylation, pTyr is relatively rare with an occurrence on only 2% of cellular proteins. Interestingly, nearly 70% of O-GlcNAcylated proteins contain at least one pTyr site^34^. O-GlcNAcylation and pTyr share many commonalities with two primary functions of both modifications being signal transduction in response to extracellular stress and stimuli as well as regulation of protein localization and complex formation across various cellular processes. Dysregulation of pTyr can lead to a variety of diseases including cancer, which has made tyrosine kinases and phosphatases promising targets for therapeutic interventions^35^. More interestingly, pTyr and the OGT Int-D appear to share similar evolutionary paths as pTyr primarily evolved in metazoans while the Int-D is only found in metazoan OGTs^36^. These parallels have long suggested cross communication between O-GlcNAcylation and tyrosine phosphorylation, but few studies have made progress in defining their interaction mechanisms.

### Ogt Int-d Is A Motif-dependent Facilitator Of Protein Interaction And O-glcnacylation

**Figure 3:**
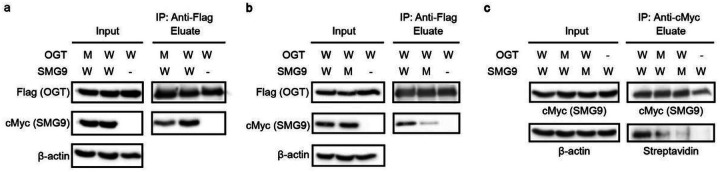
Int-D binding site is a regulator of protein association and O-GlcNAcylation. (**a**) Co-IP of cMyc-SMG9 with Flag-OGT (W) or Flag-OGT-N791A (M) from TRex-293 cells, followed by western blot detection. (**b**) Co-IP of cMyc-SMG9 (W) or cMyc-SMG9-Y147F (M) with Flag-OGT from TRex-293 cells, followed by western blot detection. (**c**) O-GlcNAcylation detection on SMG9 (W) or SMG9-Y147F (M) from cells co-expressed with Flag-OGT (W) or Flag-OGT-N791A (M). cMyc-tagged SMG9 (WT or Y147F mutant) was immunoprecipitated from TRex-293 cell lysate by cMyc-agarose, biotinylated by GalT assay, and detected by streptavidin-HRP far western blot. All whole cell lysates have endogenous OGT knocked down. Blots are representative of at least three biological replicates.

To begin investigating the biological roles of the OGT Int-D, we turned our attention to full-length protein interactions. Two primary functions facilitated by noncatalytic binding sites of enzymes are substrate recognition and non-substrate protein binding. First, we demonstrated that the OGT-SMG9 association occurs at protein level by co-immunoprecipitation (co-IP) analysis of Flag-tagged OGT and cMyc-tagged SMG9 co-expressed in human embryonic kidney 293 cells (HEK293) (Fig. 3a and b). We then selected the SMG9-Y147F mutant as well as three OGT mutants (I734R, I787E, N791A) that most effectively disrupted SMG9 peptide binding for co-IP (Fig. 3a and b, Extended data Fig. 4a and b). Compared to wild-type (WT) SMG9, the SMG9-Y147F mutant substantially decreased association with OGT, further supporting the importance of Y147 side chain in their PPI. In addition, each of the OGT mutants significantly reduced the level of SMG9 association, though none of the individual mutants completely abolished SMG9 binding. A double mutant OGT (I787E-N791A) was used to co-IP SMG9 and produced similar results (Extended data Fig. 4c). This suggests that interaction via the Int-D site is the major facilitator of OGT-SMG9 association, but may not be the only interaction between the full-length proteins. As mentioned above, our SMG9 peptide crystal structure shows that OGT residue N791 makes a bidentate interaction with the peptide backbone while residues I734 and I787 interact with hydrophobic sidechains in the peptide motif. Since all three of our tested mutants disrupt SMG9 association similarly well, we decided to continue evaluating the Int-D site with what we theorize to be a more general disrupter of OGT Int-D site binding, N791A. To evaluate the impact of Int-D site mutation on OGT’s intrinsic activity, we performed a radiolabeled activity assay. Recombinantly purified OGT or OGT-N791A were incubated with UDP-^3^H-GlcNAc and CKII peptide, one of the best OGT peptide substrates. We detected no significant difference in radio-ligand incorporation between OGT and OGT-N791A (Extended data Fig. 3c), suggesting that OGT-N791A mutant retains similar intrinsic activity as WT OGT. In our previous studies, we have used O-GlcNAcase (OGA) as a model substrate and binding partner of OGT *in vitro,* as it contains a single major O-GlcNAcylation site at S405 that is easily detected by western blot^18^. OGA does not contain the PxYx[I/L] motif, or any closely related sequence variants, suggesting that it would not be recognized by the newly discovered Int-D site. Indeed, co-IP of OGA with WT OGT and N791A mutant showed no difference in OGA association (Extended data Fig. 4d), supporting the Int-D site as a specific motif-dependent regulator of OGT protein binding.

A recent study has reported a single O-GlcNAcylation site at T114 of SMG9, 30 amino acids upstream of our identified motif^28^. The proximity of this modification site to our Int-D motif implies a coordinated substrate binding model (Extended data Fig. 2f). To evaluate changes in SMG9 O-GlcNAcylation upon disruption of Int-D site interaction, we generated TRex-293 stable cell lines expressing Flag-tagged WT OGT or N791A mutant with endogenous OGT knockdown. TRex-293 cells enable single-copy integration of exogenous constructs into an engineered genomic locus, allowing us to tightly control WT and mutant OGT expression through an inducible tet-on system. Co-expression of cMyc-SMG9 or cMyc-SMG9-Y147F with each OGT construct, followed by anti-cMyc immunoprecipitation and biotinylation of O-GlcNAc by GalT assay^37^, revealed dramatically reduced SMG9 O-GlcNAcylation in cells expressing mutants of the Int-D binding site as well as SMG9 motif (Fig. 3c). To determine if changes in O-GlcNAcylation are motif dependent, we similarly evaluated O-GlcNAcylation of Flag-tagged catalytically impaired OGA-D175N and OGT, two non-motif containing proteins, by western blot (Extended data Fig. 4e). Both proteins were comparably O-GlcNAcylated under WT OGT and N791A overexpression conditions, supporting a motif assisted recognition mode for SMG9 O-GlcNAcylation. Collectively, this study provides the first evidence of a motif dependent O-GlcNAcylation mode as well as the first evidence that the Int-D regulates OGT recognition and O-GlcNAcylation of specific substrates. Surprisingly, these results also implicate the OGT Int-D binding site as a direct facilitator of tyrosine phosphorylation crosstalk with O-GlcNAcylation. Further analysis of this novel binding site *in silico* and in cells will illuminate the extent of cross-regulation between these two important modifications.

### Pxyx[i/l] Motif-containing Proteins Participate In Diverse Cellular Processes

**Figure 4:**
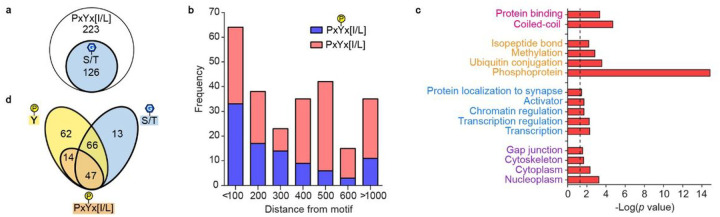
Bioinformatic analysis of PxYx[I/L] motif containing proteins. (**a**) O-GlcNAcylated proteins (blue circle), among the 223 PxYx[I/L] motif-containing proteins (white circle). (**b**) Distance of known O-GlcNAcylation sites from all PxYx[I/L] motifs (pink and blue) and phosphorylated PxYx[I/L] motifs (blue). (**c**) Gene Ontology terms associated with motif-containing proteins. Molecular function and protein domain (pink), posttranslational modifications (orange), biological processes (blue), cellular components (purple). Dotted line represents p-value cutoff of 0.05. (**d**) Overlap analysis of motif-containing proteins with reported tyrosine phosphorylation anywhere on the protein (yellow oval), O-GlcNAcylation anywhere on the protein (blue oval), and tyrosine phosphorylation on the PxYx[I/L] (orange oval).

Leveraging the SLiMSearch algorithm^38^, we performed bioinformatic analysis of the PxYx[I/L] binding motif in the human proteome, excluding peptides with proline in positions 2 or 4 and those mapped to secreted or extracellular protein domains. We identified the motif in intrinsically disordered regions (IUPred score >0.4) of 223 human proteins, 126 (56.5%) of which were found in the O-GlcNAc database with 89 proteins containing 374 assigned O-GlcNAcylation sites (Fig. 4a and Supplemental table 1 and 2)^39^. Relative to the motif, 64 (17.1%) of reported O-GlcNAcylation sites are within 100 residues, showing modest enrichment (Fig. 4b pink and blue columns). Gene ontology (GO) analysis of motif-containing proteins, using DAVID bioinformatics resources, showed significant enrichment (FDR < 0.05) across multiple cellular components and processes including transcription, transcription regulation, and chromatin regulation (Fig. 4c and Supplemental table 3)^40,41^. These processes are all governed by complex systems of PPIs and PTMs that result in wide-reaching effects across the cell. Enrichment of protein binding molecular function along other PTMs including phosphorylation, ubiquitination, and methylation, may lend further support for the Int-D motif as a regulator of cell signaling and protein complex formation.

Comparing our list of motif-containing proteins to human tyrosine phosphorylation sites reported in PhosphositePlus, we identified 189 (84.8%) proteins with at least one pTyr site (Fig. 4d and Supplemental table 2)^42^. Of these phosphorylated proteins, 61 (32.3%) are phosphorylated on our motif tyrosine, like SMG9, 47 (77.0%) of which have reported O-GlcNAcylation. Compared to all motif-containing proteins, these 47 motif phosphorylated proteins are enriched in proximal O-GlcNAcylation, with 33% of assigned O-GlcNAc modification sites falling within 100 residues of the motif (Fig. 4b blue columns). This further supports the Int-D site as a facilitator of O-GlcNAcylation cross regulation with pTyr. Interestingly, none of these motif phosphorylated proteins are receptor tyrosine kinases, pointing toward this mode of cross regulation occurring in downstream signal transduction on non-autocatalytic sites.

GO analysis of the 47 motif phosphorylated proteins demonstrated significant enrichment of only protein binding as a molecular function. Manual analysis of these proteins shows a variety of activities including kinase, phosphatase, and ubiquitin hydrolase, though the primary role of many proteins is non-catalytic binding to other proteins and protein complexes. BioGrid interaction analysis identified a set of 57 significantly enriched interactors of our motif phosphorylated protein set (FDR < 0.05) (Supplemental table 4)^43^. GO analysis of these 57 proteins showed strong enrichment of adherens and tight junction cellular components as well as other cell-cell adhesion functions (Supplemental table 5). These results suggest a role of OGT Int-D and its direct crosstalk with tyrosine phosphorylation in regulation of cell-cell adhesion as well as signaling at the cell surface.

### Ogt Int-d Binding Site Regulates Nutrient Stress Response And Lactate Production

**Figure 5:**
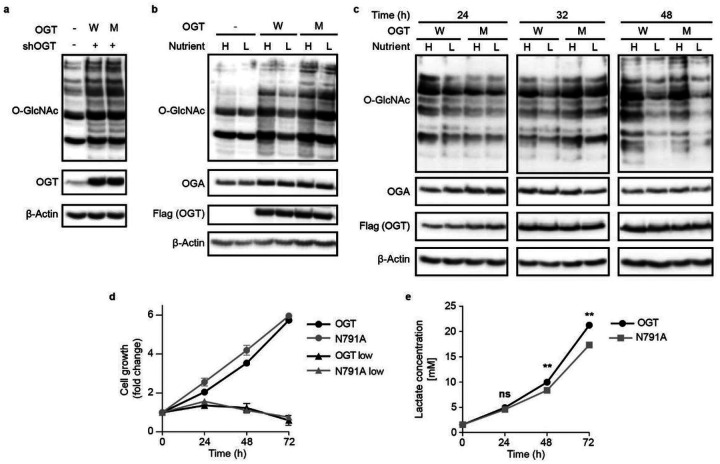
OGT Int-D binding site is a regulator of nutrient stress response and lactate production. (**a**) Western blot detection of global O-GlcNAcylation in TRex-293 cells overexpressing OGT (W) or OGT-N791A (M) with endogenous OGT knockdown (shOGT). (**b**) Western blot of O-GlcNAcylation under high (H) and low (L) nutrient conditions with OGT (W) or OGT-N791A (M) overexpression. High nutrient conditions include DMEM with 4.5 g/L glucose and 10% FBS, low nutrient conditions include DMEM with 0.45 g/L glucose and 1% FBS. (**c**) Western blot showing time course study of O-GlcNAcylation response to low nutrient stress with OGT (W) and OGT-N791A (M) overexpression. Cells were induced for 48 hours prior to high or low nutrient treatment, then samples were collected at 24, 32, and 48 hours of treatment. (**d**) Growth rate of cells overexpressing OGT (black lines) or OGT-N791A (gray lines) under high (circles) and low (triangles) nutrient conditions over 72 hours, n = 4. No significant difference was detected between cell lines in either nutrient condition. (**e**) Media lactate concentration from cells expressing WT OGT or N791A after 24, 48, and 72 hours. Lactate concentration, from LactateGlo assay, was normalized to cell number by CellTiter-Glo assay, and quantified by lactate standard curve. **p < 0.01, error bars represent standard deviation from n = 4 biological replicates. Western blots are representative of three biological replicates.

Our bioinformatic analysis showed motif-containing proteins, with and without reported O-GlcNAcylation sites, playing roles across a range of cellular processes. After establishing the Int-D site as a motif-dependent regulator of substrate specific binding and O-GlcNAcylation, we began to look at its role in cellular O-GlcNAc regulation. Previous studies have mutagenized and truncated the TPR domain to disrupt substrate binding and detected significant changes in the global O-GlcNAc profile^16–19, 44,45^. These results have established the TPR, especially those repeats closest to the catalytic domain, as broad facilitators of OGT substrate binding and O-GlcNAc modification. To assess the contribution of the Int-D binding site to the global O-GlcNAc profile, we expressed OGT and each of the Int-D site mutants in cells and evaluated O-GlcNAcylation by western blot (Fig. 5a and Extended data Fig. 5a). Unlike TPR mutations, disruption of Int-D site binding showed no obvious changes to cellular O-GlcNAc status. This supports that the motif-based substrate regulation is not a global mode of cellular O-GlcNAcylation and may play a more specific role.

An important function of the O-GlcNAc system is sensing and responding to changes in environmental nutrients and growth factors. Altered levels of glucose, amino acids, lipids, and other primary metabolites have shown to influence O-GlcNAcylation levels. Some studies posit that the HBP the pathway responsible for production of OGT’s sugar donor UDP-GlcNAc, is the primary integrator of this signaling with minimal input from OGT and OGA themselves^1^. In standard cell culture conditions, the major sources of nutrients are glucose and serum. We tested changes in cellular O-GlcNAc profile under high (4.5 g/L glucose, 10% FBS) and low (0.45 g/L glucose, 1% FBS) nutrient conditions in our TRex-293 stable cell lines. Individual glucose or serum stress from these relatively mild conditions resulted in minimal changes to O-GlcNAcylation for both WT OGT and N791A expressing cells (Extended data Fig. 5b and c). When depleted in combination, WT OGT expressing cells demonstrated a significant decrease in global O-GlcNAc signal (Fig. 5b). Surprisingly, N791A mutation appeared to desensitize the O-GlcNAcylation response to nutrient stress. A follow-up time course study revealed that N791A expression delayed the stress response (Fig. 5c). Cell growth assay showed no significant difference between the growth rate of WT OGT or N791A overexpressing cells in either nutrient condition (Fig. 5d). These results implicate the Int-D site as a temporal regulator of cellular nutrient stress signaling.

To determine if this delayed response to nutrient deprivation can be generalized to other cell lines, we stably expressed WT OGT and N791A in the cervical cancer cell line HeLa. Unlike TRex-293 cells, these cell lines only required 24 hours of low nutrient treatment to show considerable O-GlcNAc downregulation, illustrating the variability of response speed across different cell types (Extended data Fig. 6). As in our TRex-293 cells, N791A expression delayed the low nutrient stress response, relative to WT OGT expressing cells. This striking alteration in global O-GlcNAcylation response to low nutrient stress by a single OGT point mutation has not been observed in previous studies. Cellular nutrient sensing is regulated by different systems at multiple levels across the cell, explaining the eventual reduction of O-GlcNAcylation level we observed in N791A cells. It is interesting that this effect is only seen when both free glucose and serum levels are reduced, suggesting that a combination of primary metabolites and growth factors are responsible. The complex composition of serum precludes us from specifically identifying the affected signaling pathways at this point, though these results already support the Int-D binding site as an important regulator of nutrient sensing. This also supports a model of cooperative regulation between OGT and the HBP.

Response to external cues and nutrient status is only one side of OGT’s nutrient sensing role. Regulation of downstream metabolic programs, including the balance of glycolysis and oxidative phosphorylation, is a critical function of OGT^46^. Under normal conditions, healthy cells will preferentially utilize the oxidative phosphorylation pathway to metabolize glucose. However, metabolically aberrant cancer cells drive glucose consumption through both the citric acid cycle and glycolysis pathways (the Warburg effect), resulting in increased lactate production^47^. OGT overexpression has been identified in a range of clinical cancers and associated with increased glycolysis^46^. Throughout culture of our stable cell lines, we noticed rapid media acidification of WT OGT cell lines but not N791A. To evaluate differences in metabolic activity, we measured media lactate concentration in a time course using the LactateGlo assay and normalized the results to cell growth rate. Samples expressing WT OGT showed significantly higher lactate concentrations than N791A expressing cells at 48 and 72 hours (Fig. 5e), implicating the Int-D as a regulator of cellular metabolism and the Warburg effect. These simple tests of lactate production and O-GlcNAcylation response to nutrient stress have broad implications for the important roles of Int-D in health and disease, which requires further investigation.

## Discussion

Previous studies of OGT substrate regulation and protein binding have focused on the TPRs of OGT, as they are well-known structural scaffolds found in many proteins across the cell. Our study has assumed a fundamentally different route, employing the unique ProP-PD library to profile OGT surface binding sites. Our agnostic approach led to the surprising discovery of a novel motif-based binding site in the OGT Int-D. Since its initial structural characterization over a decade ago, the role of the Int-D has remained mysterious. Despite the prevalence of OGT homologs throughout the kingdoms of life, the unique fold of Int-D that intersects an otherwise typical GT-B enzyme catalytic domain is only found in metazoan OGTs. This is unlike the TPR domain whose structure and length are highly conserved across OGT homologs in prokaryotic and eukaryotic species. This may suggest that the Int-D arises with increased organismal structural and functional complexity. We have identified a unique peptide motif PxYx[I/L] that binds to the OGT Int-D site. Our observations that motif containing proteins partake in processes typically associated with evolutionarily complex functions of multicellular organisms (e.g., chromosomal structure, cell-cell adhesion, environmental sensing, etc.) support this premise. As mentioned above, tyrosine phosphorylation is also typically associated with multicellular organisms and regulation of evolutionarily complex functions. Sequence alignment of OGT Int-Ds reveals a further line of genetic delineation as the Int-D binding site residues identified in this study (e.g., F723, I734, I787, N791) are only highly conserved in vertebrates^12^. This is interesting as knockout of ogtin vertebrate systems (e.g., mouse, human) is embryonic lethal, however, *ogt* knockout is tolerated in the invertebrate *C. elegans*^48,49^. Our work leads to a hypothesis that the Int-D site is an important piece of OGT’s essential cellular functions and may be exploited for therapeutic interventions.

Hallmark phenotypes of cancer and other deleterious diseases include dysregulation of nutrient sensing and metabolism. Aberrant OGT and O-GlcNAcylation have been intimately linked to the development of cancer and other metabolic disorders. The wide reach of O-GlcNAcylation across important cellular systems makes it a valuable target for therapeutic intervention, however, disruption of these processes can also present problems. Current OGT inhibitors primarily target the active site, triggering global disruption of O-GlcNAcylation, spurring concern over unanticipated side effects. Nearly as interesting as the processes impacted by the Int-D are those that are not. Apparent maintenance of normal O-GlcNAcylation upon Int-D site mutation indicates that this binding site plays explicit roles in the cell. The structural uniqueness of the Int-D, coupled with its specific sequence recognition offer great opportunity to target OGT with exceptional specificity. Our findings for the Int-D’s role in low nutrient response and lactate production warrant further investigation of this domain’s role in cancer and metabolic disorders.

This study unifies multiple lines of thinking that have been pervasive in the field but have lacked concrete evidence: 1) major regulatory mode(s) of OGT utilize its non-catalytic regions to coordinate interactions with specific substrates; and 2) due to its evolutionary novelty, the Int-D plays one or more roles in OGT that make it distinct from other glycosyltransferases or even non-eukaryotic OGT homologs. Through multiple cellular assays we have identified the Int-D binding site as a motif-dependent regulator of protein association and O-GlcNAcylation. We further linked this site to tyrosine phosphorylation crosstalk, nutrient stress response, and metabolic regulation. Along with other important roles implicated by our bioinformatic analysis, these findings have radically changed our view of the Int-D and its importance to OGT. This opens exciting pathways to specifically dissect OGT’s regulation and functions in health and disease, creating a deluge of new opportunities in the field.

During the preparation of this manuscript, a study utilized mRNA display to screen for peptide inhibitors of OGT^50^. This screen enriched different groups of peptides including some with a similar PxYx[I/L] motif as we identified. However, the team did not investigate the binding mode of these motif-containing peptides as they did not strongly inhibit OGT’s intrinsic activity, corroborating our findings and supporting the Int-D as a novel regulatory site on OGT.

## Methods

### Proteomic peptide phage display (ProP-PD) and next generation sequencing (NGS) data analysis

Recombinantly purified OGT and OGT_4.5_ were used as bait proteins in selections against the ProP-PD library. Selections were performed following the published protocol^20^. In brief, proteins (10 μg in 100 μg PBS) were immobilized in 96 well Flat-bottom Immunosorp MaxiSorp plates for 18 h at 4 °C. Wells were blocked with 200 μg BSA (0.5% in PBS) and washed four times with 200 μg PT (PBS, 0.05% tween 20) before adding the HD2 phage library (10^11^ phages in 100 μg PBS per well), first to the GST-coated wells (1 h, 4 °C) to remove non-specific binders, and then to the bait protein-coated plates (2 h, 4 °C). Unbound phages were removed and the bound phages were eluted (100 μL log phase E coli OmniMAX, 30 min, 37 °C). M13 helper phages were added (10^9^ M13KO7 helper phages per well, 45 min at 37 °C) before transferring the bacteria to 1 mL 2xYT supplemented with 100 μg carbenicillin (Carb), 30 μg Kanamycin and 0.3 mM isopropyl^-D-1-thiogalactopyranoside (IPTG). Bacteria were grown at 37 °C for 18 h, before harvesting the phages (2,000 × g for 10 min). The phage supernatants were pH adjusted (using 1/10 volume 10x PBS) and used as in-phage for the next round of selection.

The peptide-coding regions of the naive ProP-PD library and the binding-enriched phage pools (5 μL) were PCR-amplified and barcoded using Phusion High-Fidelity polymerase (Thermo Scientific) for 22 cycles. PCR products were confirmed by 2% agarose gel electrophoresis stained with GelRed using a 50 bp marker (BioRad). PCR products were normalized using Mag-bind Total Pure NGS, pooled and purified from a 2% agarose gel (QIAquick Gel Extraction Kit), and analyzed using Illumina MiSeq v3 (1×150 bp read setup, 20% PhiX). Results were processed using in-house Python scripts. Reads were demultiplexed, adapter and barcode regions were trimmed, and sequences were translated into peptide sequences. Peptides were annotated using PepTools and assigned confidence levels based on four different criteria: occurrence in replicate selections, identification of overlapping peptide sequences, high counts, occurrence of sequences matching consensus motifs determined from the generated data set. For a stringent analysis we focused on the medium/high confidence peptides that fulfill at least three of these criteria.

### Cell Culture

Human HeLa (ATCC), HEK293T (ATCC), HEK293 (ATCC), and TRex-293 (ThermoFisher #R71007) cells were cultured in Dubelco’s modified Eagle’s medium (Corning) containing 10% fetal bovine serum (FBS) (Sigma), 10 U/mL penicillin, and 10 μg/mL streptomycin at 37°C and 5% CO_2_. Inducible knockdown of endogenous OGT in TRex-293 and HeLa cells was generated using a standard second-generation lentiviral system. Briefly, HEK293T cells were co-transfected with pCMV-VSVG, psPAX2, and EZ-Tet-pLKO-shOGT-Puro by calcium phosphate transfection. Secreted virions were collected and used to infect TRex-293 and HeLa cells. Positive clones were selected by puromycin treatment and OGT knockdown upon doxycycline induction was evaluated. TRex-293 stable cell lines were generated following the supplier’s instructions. Briefly, pcDNA5/FRT/TO plasmids containing WT OGT or OGT-N791A were co-transfected with pOG44 into TRex-293 cells with endogenous OGT knockdown and positive colonies were selected by hygromycin treatment. Various doses of doxycycline were tested in each cell line to determine conditions for equal exogenous OGT expression. HeLa stable cell lines expressing WT OGT or OGT-N791A were similarly generated by lentiviral transduction using the pLenti transfer plasmid and zeocin selection.

### Nutrient Deprivation

For nutrient treatment experiments, cells were seeded at 4.5 × 10^5^ cells/well in 6-well plates overnight then treated with normal DMEM (10% FBS, 4.5 g/L glucose) supplemented with doxycycline for 48 hours, to induce endogenous OGT knockdown and expression of our exogenous construct. Samples were then treated with the nutrient condition detailed in the results. Following low nutrient treatment, cells were washed with PBS and harvested by centrifugation. Cell pellets were lysed with RIPA total lysis buffer (150 mM NaCl, 1% nonidet-P40, 0.5% deoxycholate, 0.1% sodium dodecyl sulfate, 50 mM Tris-HCl, pH 7.4) supplemented with protease inhibitor cocktail (Millipore Sigma #P8340) and 10 μM Thiamet-G. Cell debris was removed by centrifugation at 16,000 g for 15 min at 4°C. Samples were separated on an 8% SDS-PAGE gel and detected by western blot using antibodies: anti-O-GlcNAc (RL2, ThermoFisher scientific #MA1–072), anti-OGT (Santa Cruz #sc-74546), anti-OGA (Santa Cruz #sc-376429), anti-Flag (Millipore Sigma #F1804), anti-cMyc (Millipore Sigma #C3956), and anti-β-Actin (ThermoFisher scientific #MA1–140).

### Protein Expression And Purification

Human OGT_4.5_, OGT, and mutants described above were expressed and purified as described previously^12^. Briefly, a pET24b plasmid with OGT_4.5_ gene inserted (a kind gift from S. Walker’s lab) was transformed into BL21(DE3) *E coli* for protein expression. The bacteria were cultured in LB medium supplemented with 50 μg/mL kanamycin at 37°C, 250 rpm. After reaching to an OD_600_ of 0.6–0.8, the 0.3 mM IPTG was added to induce protein expression at 16°C, 220 rpm overnight. Bacterial cells were collected and resuspended in TBS buffer (150 mM NaCl, 20 mM Tris, pH 8.0) supplemented with 1 mM phenylmethylsulfonyl fluoride. The suspension was lysed by pressure cell homogenizer, clarified by centrifugation, and subjected to Ni-NTA affinity chromatography. The eluted OGT_4.5_ protein was incubated with HRV-3C protease to cleave the N-terminal His_6_-tag overnight. The protein sample was subsequently purified by a size exclusion chromatography column (Superdex 200 increase 10/300; Cytiva) on an AKTA FPLC system in TBS buffer with 0.5 mM tris(3-hydroxypropyl)phosphine. OGT_4.5_ protein was concentrated to 8 mg/mL for crystallization.

### Ciystallization

All peptides for crystallization were prepared by solid phase peptide synthesis (Ontores, ≥ 95% purity, HPLC). Hanging drop vapor diffusion method was used for co-crystallization. OGT_4.5_ was first incubated with 2 mM of peptide and 1 mM of UDP-GlcNAc on ice for two hours. After pre-incubation, 2 μL of protein complex was mixed with 1 μL of reservoir solution and equilibrated against 200 μL of reservoir solution at 20°C. OGT_4.5_:UDP-GlcNAc:SMG9 and OGT_4.5_:UDP-GlcNAc:ZNF831 crystals were obtained in the condition consisting of 1.9 M ammonium sulfate, 1 % xylitol, 0.1 M Tris, pH 8.5, while OGT_4.5_:UDP-GlcNAc:CP37 crystals were grown in the condition containing 1.45 M potassium phosphate, 1 % xylitol, 10 mM EDTA, pH 8.0. Crystals were cryo-protected in mother liquor supplemented with 27% xylitol and then flash frozen in liquid nitrogen.

### X-ray Data Collection, Processing, And Structure Determination

All the X-ray data were collected on the Life Sciences Collaborative Access Team (LS-CAT) beam lines 21-ID-F (for OGT_4.5_:UDP-GlcNAc:SMG9) and 21-ID-D (for OGT_4.5_:UDP-GlcNAc:ZNF831 and OGT_4.5_:UDP-GlcNAc:CP37) in Argonne National Laboratory, IL. Data were processed using autoPROC^51^, which is a toolbox containing programs XDS and AIMLESS for data indexing and scaling^52,53^. Structures were solved by molecular replacement with Phaser using OGT_4.5_ (PDB 3PE3) as a search model^12,54^. Iterative model building was performed in COOT^55^, followed by refinement with PHENIX^56^. OGT_4.5_:UDP-GlcNAc:SMG9 and OGT_4.5_:UDP-GlcNAc:ZNF831 crystals belong to the space group P321, while OGT_4.5_:UDP-GlcNAc:CP37 crystal belongs to space group H3. All of these crystals contain four molecules in each asymmetrical unit, with each OGT_4.5_ in complex with one molecule of peptide and UDP-GlcNAc. All the structural figures were prepared using PyMOL (The PyMOL Molecular Graphics System, Version 2.0 Schrodinger, LLC).

### Microscale Thermophoresis (Mst)

5-FAM labeled SMG9 peptide was prepared by solid phase peptide synthesis (Ontores, ≥ 95% purity, HPLC). Two-fold serial dilutions of OGT in TBS buffer supplemented with 0.05% tween 20 was prepared in strip-tubes, and then mixed with equal volume of 100 nM of 5-FAM SMG9 peptide in the same buffer. After 10 min pre-incubation, microscale thermophoresis upon OGT-peptide binding was measured in standard capillaries on Monolith NT.115 pico instrument (NanoTemper). MST was performed with three biological replicates. Dose-response curves were plotted, and the *K*_d_ values were calculated using Origin.

### Thermal Shift Assay (Tsa)

Thermal shift assay was carried out on a StepOnePlus Real-Time PCR System (Applied Biosystems). Reactions were set up in 96-well qPCR plates with optical seal at a total volume of 20 μL. Each reaction contained 2.5 μM protein, 3.2x SYPRO Orange dye (ThermoFisher, Cat# S6650) and 200 μM peptide in TBS buffer. At least three replicates were prepared for each reaction. Using the melt curve setting fluorescent readings were collected at 0.5°C intervals from 25–95°C using the ROX channel. Melting curves were plotted in StepOne software and the first derivatives of the fluorescence curves were used to determine T_m_ values.

### Plasmids And Cloning

The original OGT_4.5_, OGT, and OGA-D175N constructs, cloned into bacterial expression vectors, have been described previously. OGT cDNA, with codon optimization for mammalian expression, was provided by Harvard PlasmID (HsCD00045640). pDONR221-SMG9 was obtained from the DNASU plasmid repository (HsCD00533575). Mammalian OGT, OGA-D175N, and SMG9 were subcloned into pcDNA5/FRT/TO (Millipore Sigma) with an added N-terminal Flag tag on OGT and OGA and an N-terminal cMyc tag on SMG9. Site-directed mutagenesis was used to generate OGT mutants for bacterial and mammalian expression as well as the SMG9-Y147F mutant construct. Short hairpin RNA (shRNA) for endogenous human OGT knockdown was designed using the RNAi Consortium shRNA library and cloned into EZ-TET-pLKO-Puro (Addgene #85966). For viral transduction, Flag-tagged OGT constructs were cloned into the pLenti vector which was a kind gift from M. B. Jackson. Viral packaging and envelope plasmids pCDH and pVSVG were a kind gift from X. Zhao. All primers used in this study are listed in Supplemental Table 6.

### Fluorescence Polarization (Fp) Assays

All FP measurements were carried out in black small volume 384-well plates (Greiner Bio-One). For saturation binding assays, 2-fold serial dilutions of recombinantly purified OGT were prepared in TBS (150 mM NaCl, 20 mM Tris-HCl, pH 8.0). 5-FAM-SMG9 fluorescent peptide at 0.4 μM in TBS was added in equal volume to the OGT dilution series. For competition binding assays, 2-fold serial dilutions of unlabeled peptide were prepared in TBS. An equal volume of 20 μM OGT with 0.4 μM 5-FAM-SMG9 in TBS was added to the dilution series. After 20 min incubation in the dark at room temperature, FP was read on a BMG PheraStar multimode plate reader equipt with 488, 520, 520 FP filter cube (BMG Labtech).

### Immunoprecipitation (Ip)

For OGT-SMG9 co-immunoprecipitation experiments, HEK293 cells were co-transfected with the indicated protein constructs or an empty vector by calcium phosphate transfection. After 48 hours of transfection, cells were washed with PBS and collected by centrifugation. Pellets were gently lysed using IP lysis buffer (150 mM NaCl, 1 % nonidet-P40, 20 mM Tris-HCl, pH 7.4) supplemented with protease inhibitor cocktail and 10 μM Thiamet-G. Total cell lysate was diluted to 1 mg/mL and incubated with Flag M2 agarose (Millipore sigma #A2220) for 16 hours at 4°C. Samples were washed four times with ten bed volumes of IP lysis buffer and eluted by boiling with 2x SDS loading buffer. Samples were run on 8% SDS-PAGE gels and detected by western blots.

### O-glcnac Detection By Galt Assay

SMG9 immunoprecipitation for O-GlcNAc detection was carried out similarly to the above co-IP and GalT assisted biotinylation was performed as previously described^37^. Briefly, TRex-293 cells stably expressing the indicated OGT constructs were transfected with the indicated SMG9 construct by calcium phosphate transfection. 48 hours after transfection, cells were treated with 10 μM Thiamet-G for 16 hours to inhibit O-GlcNAc hydrolysis, allowing O-GlcNAc accumulation on SMG9. Cells were collected, lysed, and loaded on cMyc agarose as described above. Samples were washed four times with ten bed volumes of IP lysis buffer then eluted twice with soft elution buffer (0.2% SDS, 0.1 % tween 20, 50 mM Tris-HCl, pH 8.0) for 10 min at 35°C. Samples were precipitated with methanol at −20°C overnight and spun down at 16,000 g for 30 min at 4°C. Protein pellets were reconstituted in GalT buffer (0.6% nonidet-P40, 5 mM MnCl_2_, 150 mM NaCl, 20 mM HEPES, pH 7.5) with 25 μM uridine diphosphate Nazidoacetylgalactosamine (UDP-GalNAz) and 2.2 μM GalT-Y289L and incubated overnight at 4°C. To biotinylate our modified SMG9, we used copper assisted click chemistry to add a Biotin-PEG4-Alkyne (Click Chemistry Tool #TA105–100) probe to the azide functionalized GalNAc residues. Samples were run on 8% SDS-PAGE gel and analyzed by far western blot with streptavidin-HRP (Cytiva #RPN1231) as well as western blot with cMyc and β-Actin antibodies mentioned above.

### Radiolabeled Activity Assay Of Ogt

Purified OGT (500 nM of wild-type or N791A mutant) was incubated with 50 μM UDP-^3^H-GlcNAc (specific activity 0.3 Ci/mmol, PerkinElmer #NET434250UC) and 500 μM CKII peptide (YPGGSTPVSSANMM) in the reaction buffer (20 mM Tris pH 8.0, 150 mM NaCl, 0.5 mM THP) at 37°C for 3 hours. Reactions were quenched by spotting the samples onto Amherst Protran 0.1 μm nitrocellulose membrane (Cytiva), air-dried, and washed for 5 min four times in PBS buffer. The radioactivity of each membrane was counted by a Tri-Carb 2900 TR Liquid Scintillation Analyzer (PerkinElmer). A reaction without OGT was set up as negative control. Another reaction without the washing steps was counted as a total of 50 μM of UDP-GlcNAc input to calculate the O-GlcNAcylation level of CKII peptide in each reaction. The experiment was performed in triplicate.

### Cell Growth Assay

Cell viability was assessed using CellTiterGlo 2.0 reagent (Promega) according to the manufacturer’s instructions. Briefly, cells were seeded in 96-well tissue culture plates, with at least four biological replicates for each condition and time point. Cells were grown in the indicated nutrient condition for the specified amount of time before an equal volume of CellTiterGlo 2.0 reagent was added directly to the well and incubated at room temperature for 10 min in the dark. Luminescence was measured on a BioTek Synergy H1 plate reader.

### Lactate Production Assay

Lactate production was measured using the LactateGlo assay kit (Promega) following manufacturer’s instructions. Briefly, cells were seeded in 96-well tissue culture plates with at least four biological replicates for each time point. At the specified time point, a sample of culture media was taken and diluted 1000x fold in PBS. A lactate standard curve from 0.2–200 μM in PBS was also prepared to quantify media lactate concentration. Samples were mixed with LactateGlo detection solution in low volume black 384-well plates and incubated at room temperature in the dark for 1 hour. Luminescence was measured on a BioTek Synergy H1 plate reader. Lactate concentration was normalized to cell number using the above cell growth assay protocol.

### Statistical analysis

All of the data shown are mean values with error bars representing standard deviation. Statistical significance was determined using two-tailed student’s t-test.

## Data Availability

Atomic coordinates of the OGT_4.5_ co-crystal structures have been deposited in RCSB Protein Data Bank under accession codes 8FE6 (OGT_4.5_:UDP-GlcNAc:CP37), 8FE7 (OGT_4.5_:UDP-GlcNAc:SMG9), and 8FUF (OGT_4.5_:UDP-GlcNAc:ZNF831). Data will be made available upon reasonable request to the corresponding author.
